# Dynamics of single photon transport in a one-dimensional waveguide two-point coupled with a Jaynes-Cummings system

**DOI:** 10.1038/srep33867

**Published:** 2016-09-22

**Authors:** Yuwen Wang, Yongyou Zhang, Qingyun Zhang, Bingsuo Zou, Udo Schwingenschlogl

**Affiliations:** 1 Beijing Key Lab of Nanophotonics & Ultrafine Optoelectronic Systems and School of Physics, Beijing Institute of Technology, Beijing 100081, China; 2 King Abdullah University of Science and Technology (KAUST), Physical Science and Engineering Division (PSE), Thuwal 23955-6900, Saudi Arabia

## Abstract

We study the dynamics of an ultrafast single photon pulse in a one-dimensional waveguide two-point coupled with a Jaynes-Cummings system. We find that for any single photon input the transmissivity depends periodically on the separation between the two coupling points. For a pulse containing many plane wave components it is almost impossible to suppress transmission, especially when the width of the pulse is less than 20 times the period. In contrast to plane wave input, the waveform of the pulse can be modified by controlling the coupling between the waveguide and Jaynes-Cummings system. Tailoring of the waveform is important for single photon manipulation in quantum informatics.

In recent years much attention is paid to the photon transmission and correlation in one-dimensional waveguides coupled with a wide variety of quantum systems, as such structures have important potential applications^1^,^2^. The quantum systems include dots^2^,^3^,^4^, optomechanical cavities^5^, single or multiple atoms with two or multiple levels^6^,^7^,^8^,^9^,^10^,^11^, and cavities with an atom^12^,^13^ or a Kerr medium^14^ inside. Many integrated systems have been designed in this context^15^,^16^,^17^, for which electromagnetically induced transparency^18^,^19^,^20^,^21^, Fano resonance^22^,^23^,^24^, polarization effects^1^, slow light behavior^19^,^25^, nanocavity entanglement^26^, and multi-photon transmission^27^,^28^,^29^ have been studied. Scattering of multi-photon states by a quantum system can induce correlations among the photons^30^,^31^,^32^,^33^. Going beyond local coupling by means of *δ* functions^34^, non-*δ* coupling between the waveguide and a side cavity has been considered in ref. 35 to demonstrate the influence on the single photon transmission, which extends potential applications of waveguides coupled with quantum systems.

For simplicity, it is generally assumed that the incident light is a plane wave, while in optical devices and quantum information processing the signals are typically pulses. It therefore is important to study the single photon transport for pulse input as well as the related time-dependent dynamics^32^,^33^ (which are of little interest for plane wave input). As they play a key role in single photon transport^35^, we will consider non-*δ* coupling effects between the waveguide and side cavity for the two structures shown in Fig. 1: straight and bended waveguides coupled with side cavities containing a two-level atom^8^,^9^,^11^. The cavity and two-level atom together are described by a Jaynes-Cummings system, whose modes can hybridize with the waveguide modes to form hybridization states. For the bended waveguides used in experiments^36^,^37^, the coupling to the cavity can be strongly non-local. In this work, we assume that the curvature is small, since in sharply bended waveguides the modes are modified^38^,^39^. We use a real-space Hamiltonian to find the dynamical equations for single photon waves and derive analytical expressions for the transmissivity and the hybridization states. We numerically evaluate the dependence of the hybridization state energies on the coupling between the waveguide and Jaynes-Cummings system and compare the transmission spectra for plane waves and pulses. Moreover, the dynamical behaviour is discussed in detail for a Gaussian pulse.

## Results

**Figure 1:**
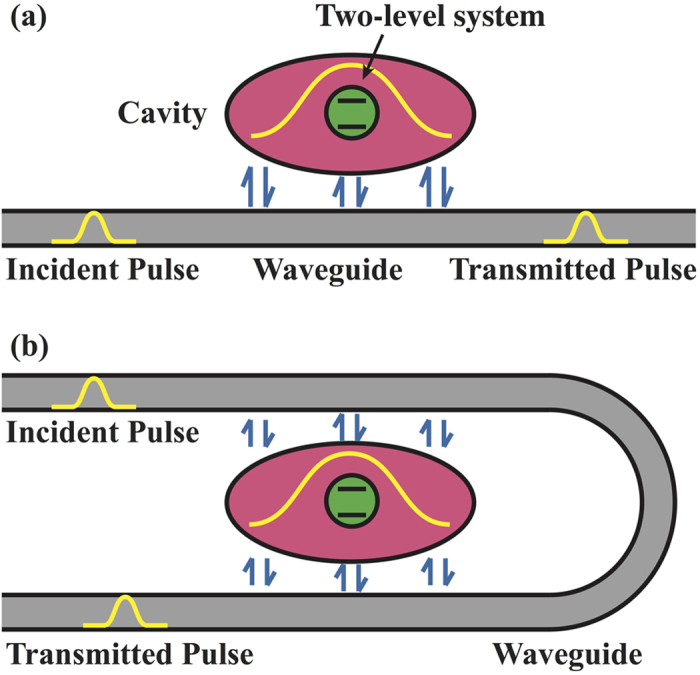
Schematics of (**a**) straight and (**b**) bended waveguides coupled with Jaynes-Cummings systems. Arrows indicate the photon tunnelling between the side cavity and waveguide.

Assuming that the two-level quantum system contained in the cavity, see Fig. 1, is a two-level atom, we first derive the dynamic equations. The real-space Hamiltonian that describes the single photon transport can be written as^35^,^40^


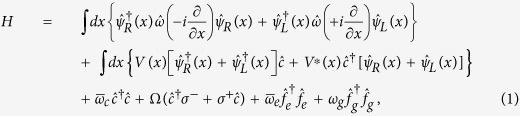


where 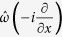 and 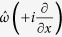 are the energy operators of the right-moving, 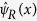, and left-moving, 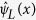, waveguide photons, respectively, 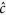 and 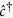 are the annihilation and creation operators of the cavity photons with energy 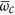, 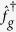 (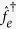) is the creation operator of the atomic ground (excited) state, and 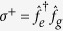 (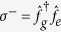) is the atomic raising (lowering) operator. The ladder operators satisfy the relations 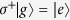, 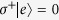, 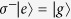, and 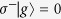, where |*g*〉 and |*e*〉 are the atomic ground and excited states with energies *ω*_*g*_ and 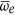 (so that the atomic transition energy is 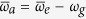). *V*(*x*) and Ω describe the coupling of the cavity photon with the waveguide mode and two-level system, respectively. Note that 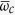 and 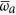 are complex numbers if we consider losses of the cavity and two-level system (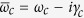 and 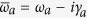 with transition energies *ω*_*c*_ and *ω*_*a*_ and losses *γ*_*c*_ and *γ*_*a*_). Throughout this work, the Plank constant *ħ* is set to be 1 for simplicity.

For single photon transport we can expand the single occupation state of the system as


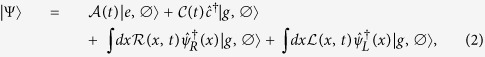


where 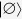 indicates that there is no photon present in the waveguide and cavity, 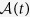 and 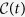 are the excitation amplitudes of the atom and cavity, and 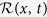 and 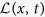 are the wave functions of the right- and left-moving waveguide photons, respectively. From the Schrödinger equation 
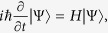
 we obtain a set of dynamic equations for 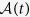, 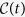, 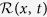, and 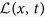 as


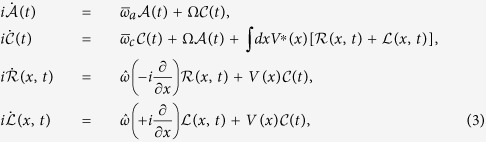


where dots denote first-order derivatives with respect to time. The energy operators can be linearized around the energy of the cavity photon as^40^


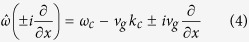


by introducing a group velocity *v*_*g*_ and a wave vector *k*_*c*_. Equations (3) and (4) together describe the dynamics of the single photon transport in the waveguide.

Using the approach of ref. 35, which is valid when the dispersion of the waveguide mode can be linearized near the cavity energy *ω*_*c*_, we obtain


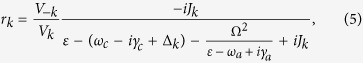



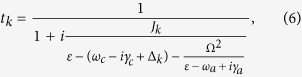


for plane wave input, where 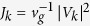, *r*_*k*_ and *t*_*k*_ are the reflection and transmission coefficients, respectively, and *ε* and *k* are the energy and wave vector of the incident photon. 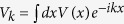 is the Fourier transform of *V*(*x*) and


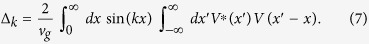


For 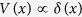 our *t*_*k*_ agrees with the results of previous works^25^,^40^. The transmissivity of the plane wave is given by 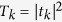 and that of a pulse by


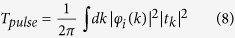


where 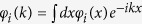 and 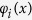 is the wave function of the incident pulse.

The coupling between the waveguide and Jaynes-Cummings modes leads to two branches of hybridization states. When the energy of the incident photon equals that of a hybridization state the transmissivity is minimal. The energies of the upper and lower hybridization states are


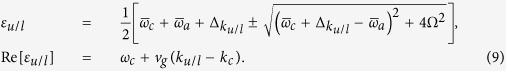


For *δ* coupling [*V*(*x*) ∝ *δ*(*x*)] between the waveguide and Jaynes-Cummings modes, we have 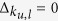, refer to Eq. (7), so that we obtain 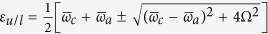, i.e., the energies of the Jaynes-Cummings modes, while non-*δ* coupling can shift the hybridization states. The Hamiltonian in Eq. (1) is linearized near the energy of the cavity mode and thus valid only when the photon wave vector is near *k*_*c*_. In this case we have 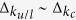.

We consider two Gaussian-type coupling functions


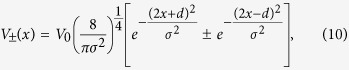


where *σ* is the width of the Gaussians, *d* their separation, and *V*_0_ measures the coupling strength between the waveguide and cavity. Both coupling functions are suitable for the bended waveguide (and, of course, also for the straight waveguide). For 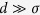 we have two-point coupling with the cavity. The fact that *V*_+_(*x*) is even and *V*_−_(*x*) is odd corresponds to the even and odd parities of the cavity modes. In the following the units of the energy/frequency, wave vector, length, and time are taken as *ω*_*c*_, *k*_*c*_, 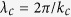, and 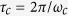, respectively.

**Figure 2:**
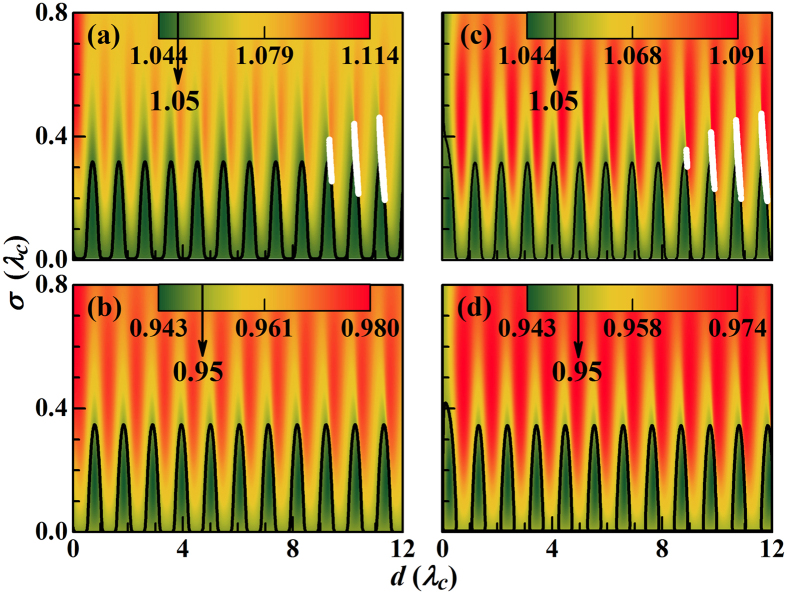
Contour maps of the energies of the hybridization states as functions of *d* and *σ*. (**a**) *ε*_*u*_ for *V*_+_(*x*), (**b**) *ε*_*l*_ for *V*_+_(*x*), (**c**) *ε*_*u*_ for *V*_−_(*x*), and (**d**) *ε*_*l*_ for *V*_−_(*x*). The white regions in (**a**,**c**) indicate that there are three hybridization states in the interval 1.05 ± 0.05 *ω*_*c*_. In the upper and lower panels the black contour lines denote the levels 1.05 *ω*_*c*_ and 0.95 *ω*_*c*_ (energies of the Jaynes-Cummings modes), respectively. Parameters: *ω*_*a*_ = *ω*_*c*_, *V*_0_ = 0.1 *ω*_*c*_, Ω = 0.05 *ω*_*c*_, 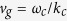, 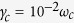, and 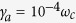.

Since the hybridization states determine the single photon transmission and dynamics, we calculate the dependence of their energies on the coupling between the waveguide and Jaynes-Cummings system. Substituting Eq. (7) into Eq. (10) results in Fig. 2. For 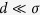 (single point coupling) the energies of the hybridization states for even coupling are always blueshifted with respect to the Jaynes-Cummings modes, while for odd coupling there is first a redshift and then a blueshift with increasing *σ*, consistent with ref. 35. The energies of the hybridization states periodically shift with the separation between the coupling points, due to a periodical dependence of Δ_*k*_ on *d*. This period is half of that of the Fourier transforms


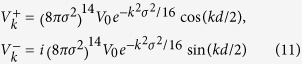


of *V*_±_(*x*), i.e., 4*π*/*k*. As a result, the period of *ε*_*u*_ is 
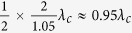
 [see Fig. 2(a,c)] and that of *ε*_*l*_ is 
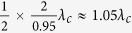
 [see Fig. 2(b,d)]. Because Δ_*k*_ also periodically varies with *k* (or *ε*) and this period decreases with increasing *d*, more than one hybridization state is expected for certain values of *d* and *σ*. The numerical results show that there are three hybridization states in the interval 1.05 ± 0.05 *ω*_*c*_ in the white regions in Fig. 2(a,c). This multi-solution property can strongly influence the spectral form of the single photon transmission, see below.

**Figure 3:**
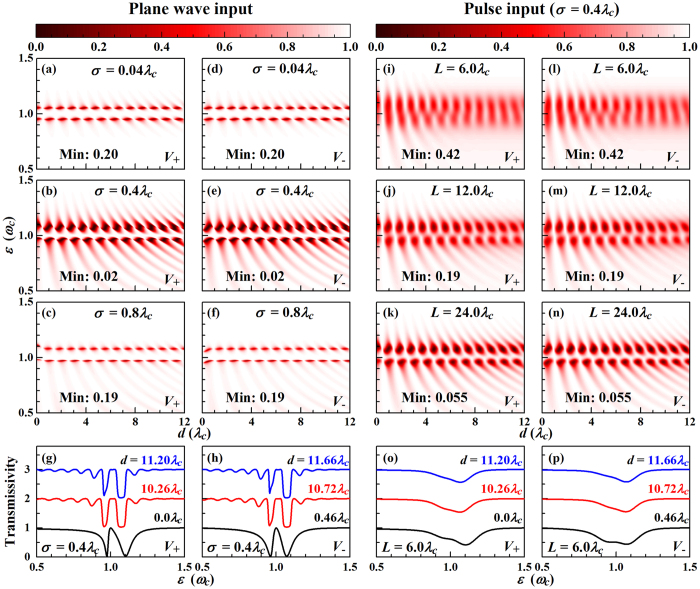
Contour maps of the transmissivity of the single photon (**a**–**f**) plane wave and (**i**–**n**) pulse as functions of *d* and *ε*. Transmission spectra for the (**g**,**h**) plane wave and (**o**,**p**) pulse for different values of *d*, offset in steps of 1. Except those denoted in the figures, we use the parameters of Fig. 2.

Figure 3 shows the transmission spectra of the single photon plane wave and pulse. The periodical variations of the hybridization state energies are reflected by a periodical dependence on *d* with period 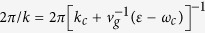. For increasing *ε* the transmission period decreases, see the arc-like patterns in Fig. 3(a–f,i–n). The parameter 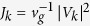 for *V*_+_(*x*) reaches its maximum value of 
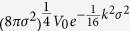
 for *kd* = 2*nπ* (*n* is an integer) and zero for *kd* = (2*n* + 1)*π*. For *V*_−_(*x*) those two branches are exchanged. Thus, for *d* → 0 there are two transmission dips for *V*_+_(*x*), while the transmissivity always equals 1 for *V*_−_(*x*). The latter implies that the waveguide mode decouples from odd cavity modes for local coupling. For 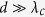 the influence of the oscillation of 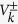 as a function of *k* (or *ε*) becomes stronger. Since Δ_*k*_ and *J*_*k*_ are periodic functions in *k* [Eqs (6), (7) and (11)] with periods decreasing for increasing *d*, the transmission oscillates faster and faster when *d* increases, see Fig. 3(a–f). According to Fig. 3(a–f), the influence of the Jaynes-Cummings system first increases and then decreases for increasing *σ* for both coupling functions, following the behaviour of 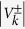, which is maximal with a value of 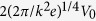 at *σ* = 2*/k*. For *k* ~ *k*_*c*_ the transition value of *σ* is about 0.32*λ*_*c*_, so that Fig. 3(b,e) refer to strong coupling (*σ* = 0.4*λ*_*c*_). On the other hand, the two transmission dips near *ε*_*c*_ represent resonant coupling between the waveguide and Jaynes-Cummings modes. Therefore, their energies are determined by the hybridization states. For values of *d* and *σ* in the white regions in Fig. 2(a,c) the transmission dips near 1.05 *ω*_*c*_ become rectangular, see Fig. 3(g,h), as there exist three solutions for the upper hybridization states. For comparison, the transmission spectra for *V*_+_(*x*) with *d* = 0 and *V*_−_(*x*) with *d* = 0.46*λ*_*c*_ are also plotted in Fig. 3(g,h). The asymmetry of the transmission dips in these cases is due to the non-*δ* coupling between the waveguide and cavity^35^.

Figure 3(i–n) show the transmissivity of the incident Gaussian pulse


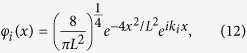


where *L* is the pulse width, *k*_*i*_ is the center wave vector, and 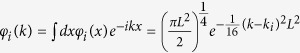 is the Fourier transform. We note that the linearization in Eq. (4) is valid for an ultrafast pulse with *L* = 6*λ*_*c*_, since we have the probability 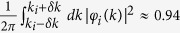 for *δk* = 0.1*k*_*c*_. In order to achieve strong coupling between the waveguide and Jaynes-Cummings modes, the width of the coupling functions is set to *σ* = 0.4*λ*_*c*_. For an ultrafast pulse (with a duration of several oscillation periods), see Fig. 3(i,l), the transmissivity does not approach zero, as many plane wave components are contained in the pulse and only a fraction is resonant. For increasing width the amount of non-resonant components decreases and the transmissivity starts approaching that of the centre mode of the pulse, see Fig. 3(i–k,l–n), where the transition point is *L* ~ 20*λ*_*c*_. The effects of the multi-solutions for the hybridization states are suppressed by the fact that there are many plane wave components in the pulse. The transmission dips due to the upper hybridization states are no longer rectangular for the ultrafast pulse, see Fig. 3(o,p). Since the functions in Eq. (11) are periodic in *d*, the transmission is periodic whether the cavity contains a two-level atom or not. However, without atom we obtain only one anti-resonant transmission peak instead of two. We note that ultrafast laser pulses can be achieved experimentally, such as a 5 fs laser pulse with a centre wavelength of 800 nm^41^. For plane wave input it is known that the transmitted wave shows only a phase difference (determined by the transmission coefficient), so that the transmissivity determines all the transport properties, while for pulse input the transmitted wave, in general, is modified. Accordingly, the transmissivity does not fully reflect the transport characteristics, so that studying the transport dynamics is necessary for single photon pulses.

The transport dynamics are determined from Eq. (3). Because the coupling between the waveguide and cavity depends on the position, it is difficult to find the solutions for *R*(*x*, *t*) and *L*(*x*, *t*) near the cavity. However, we are interested in the transmitted and reflected pulses


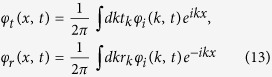


where *φ*_*i*_(*k*, *t*) is the Fourier transform of the incident pulse *φ*_*i*_(*x*, *t*), using *r*_*k*_ and *t*_*k*_ from Eqs (5) and (6). We study the Gaussian pulse


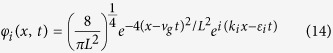


with width *L*, where *k*_*i*_ denotes the center wave vector and 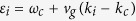. Before it meets the cavity the incident pulse satisfies the wave equation 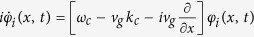.

**Figure 4:**
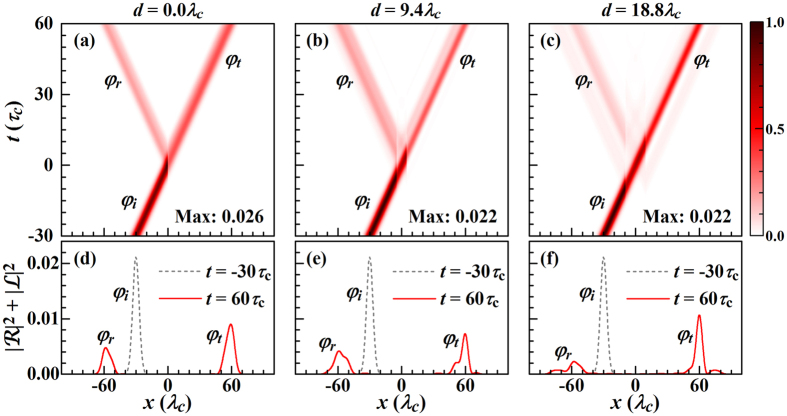
(**a**–**c**) Contour maps of the photon probability densities (normalized to their maximum values, given in the maps) as functions of *x* and *t*. (**d**–**f**) Probability distributions of the incident (*φ*_*i*_), reflected (*φ*_*r*_), and transmitted (*φ*_*t*_) pulses. The figure refers to 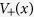 using *σ* = 0.4*λ*_*c*_, *ε*_*I*_ = 1.05 *ω*_*c*_, *L* = 12*λ*_*c*_ and otherwise the parameters of Fig. 2.

Figure 4 shows the time evolution, indicating the incident, reflected, and transmitted pulses. Without loss of generality we consider only 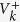, as 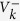 differs only by a phase factor, see Eq. (11). *V*_+_(*x*) represents for *d* = 0 the one-point coupling between the waveguide and cavity. The time evolution of the photon probability density in Fig. 4(a) and the corresponding density distributions of the incident, reflected, and transmitted pulses in Fig. 4(d) show that when the Gaussian pulse meets the side cavity it is partly reflected and transmitted. The shapes of the reflected and transmitted pulses are no longer Gaussian. Recall that for plane wave input the shape was conserved. The reflected and transmitted pulses, on the other hand, can be tailored by controlling the coupling between the waveguide and cavity. For the two-point coupling (d >> σ) we study 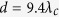. The reflected and transmitted pulses have no longer Gaussian shape and satellite peaks appear, see Fig. 4(b,e). When the incident pulse meets the left coupling point it partially continues along the waveguide, partially is stored in the Jaynes-Cummings system, and partially transfers to the second coupling point, which is the origin of the satellite peaks. With increasing *d* the influence of the above processes on the reflected and transmitted waves becomes more obvious, as shown in Fig. 4(c,f). In Fig. 4(f) the highest peak corresponds to the process that the photon continues along the waveguide, the long tail is due to the temporary storage of the photon in the Jaynes-Cummings system, and the hump next to it originates from the photon transfer. The latter becomes more apparent for *d* > *L*, compare Fig. 4(e,f). The photon transfer from the left to the right coupling point induces a group advancement, but the temporary storage in the Jaynes-Cummings system leads to a group delay. This group delay hardly depends on *d* but on the coupling between cavity and two-level atom. Because the loss typically is far less for a two-level atom than a cavity, the group delay can be enhanced by adding the atom. The group advancement, on the other hand, is less influenced by the two-level atom and appears only for *d* > *L*, which is useful for tailoring the shapes of the reflected and transmitted pulses.

**Figure 5:**
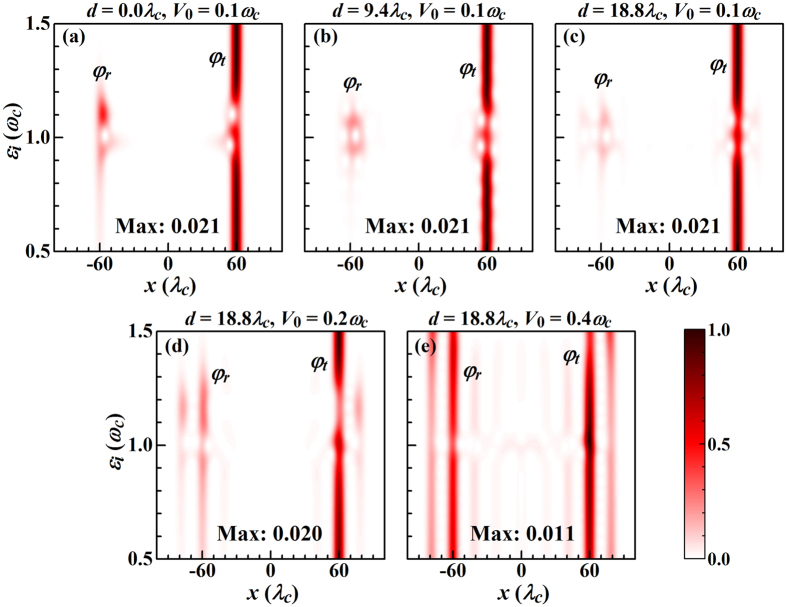
Contour maps of the probability distributions of the reflected and transmitted pulses (normalized to their maximum values, given in the maps) as functions of *x* and *ε*_*i*_. The figure refers to *V*_+_(*x*) using *σ* = 0.4*λ*_*c*_, *ε*_*I*_ = 1.05 *ω*_*c*_, *L* = 12*λ*_*c*_, *t* = 60*τ*_*c*_, and otherwise the parameters of Fig. 2.

According to Fig. 5, the reflected and transmitted pulses show a strong dependence on *ε*_*i*_, especially when *ε*_*i*_ ~ *ω*_*c*_. They obviously are not Gaussian and depend not only on *d*. For *ε*_*i*_ ~ *ω*_*c*_ in Fig. 5(a–c) weak satellite peaks develop from the main peaks (centered at *x* = ±60*λ*_*c*_) for increasing *d*. The effects of photon transfer between the two coupling points and temporary photon storage in the Jaynes-Cummings system are weak as compared to the photon transport along the waveguide for small *V*_0_ = 0.1 *ω*_*c*_. When the coupling strength between the waveguide and cavity is increased to *V*_0_ = 0.4 *ω*_*c*_ the situation changes, see, for example, the regions near 1.2 *ω*_*c*_ in Fig. 5(d) and 1.4 *ω*_*c*_ in Fig. 5(e). Moreover, for *d* > *L* all satellite peaks are clearly separated from the main peaks, demonstrating that the Gaussian shape can be divided into multiple peaks in the reflected and transmitted pulses, i.e., the waveform of the single photon pulse can be tailored. This fact also indicates that the transmissivity is not enough to describe the pulse transport in a waveguide coupled with a quantum system. For designing an ultrafast single photon device thus more attention should be paid to the pulse dynamics, including the waveforms of the transmitted and reflected pulses.

**Figure 6:**
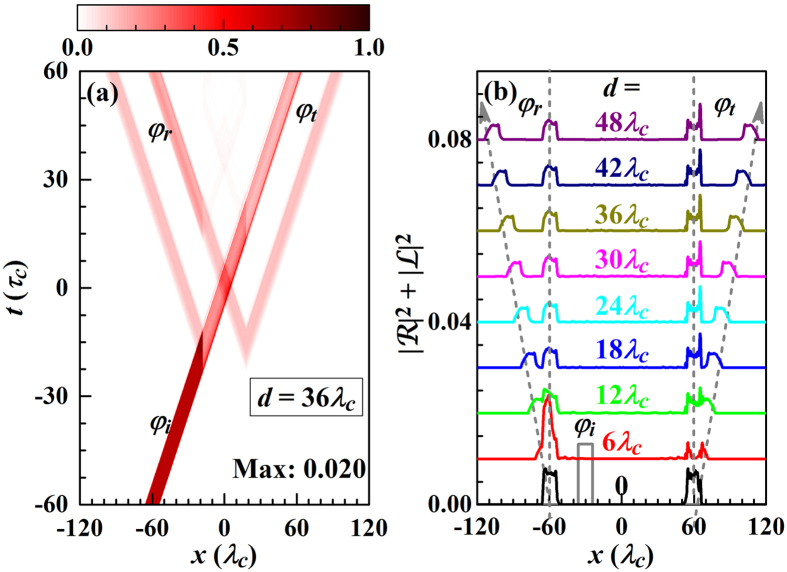
(**a**) Contour map of the photon probability density (normalized to its maximum value 0.020) as functions of *x* and *t* for an incident square pulse [grey solid line in (**b**)]. (**b**) Probability distributions of the reflected and transmitted pulses for different *d*. The figure refers to *V*_+_(*x*) using *σ* = 0.4*λ*_*c*_, *L* = 12*λ*_*c*_, *V*_0_ = 0.2 *ω*_*c*_, *ε*_*I*_ = 1.15 *ω*_*c*_, *t* = 60*τ*_*c*_, and otherwise the parameters of Fig. 2.

To demonstrate that the proposed structure is effective also for other types of pulses, Fig. 6(a) shows the time evolution of the probability distribution for an incident square pulse. For *d* > *L* the transmitted pulse is divided into two main parts, the right part originating from the photon transfer between the two coupling points and the left part from the transport along the waveguide. When *d* increases the separation between the two parts increases, see Fig. 6(b). Moreover, since the group advancement is influenced only weakly by the two-level atom, see above, the situation remains similar for a cavity without atom. The two-point coupling structure of Fig. 1(b) has great potential in single photon manipulation in quantum informatics.

## Discussion

We have studied the dynamics of a single photon pulse in a one-dimensional waveguide two-point coupled with a Jaynes-Cummings system. The transport is described by a set of dynamic equations, derived from a real-space Hamiltonian. For an ultrafast Gaussian pulse we have shown that the energies of the hybridization states between the waveguide and Jaynes-Cummings modes periodically shift with the separation between the two coupling points. They can be blueshifted or redshifted with respect to the energies of the Jaynes-Cummings modes. For both plane wave and pulse inputs the transmissivity also depends periodically on the separation between the two coupling points. When the duration of the Gaussian pulse is about 20 times the oscillation period the transmissivity approaches that of the centre plane wave. It is known for plane waves that the shapes of the reflected and transmitted waves are identical to that of the incident wave, while this rule generally does not apply to pulses. For Gaussian (and square) pulses we have demonstrated multi-peak shapes by increasing the separation between the two coupling points. As a result, we provide an effective method to adjust the single photon waveform in ultrafast photonics.

## Additional Information

**How to cite this article**: Wang, Y. *et al*. Dynamics of single photon transport in a one-dimensional waveguide two-point coupled with a Jaynes-Cummings system. *Sci. Rep.* **6**, 33867; doi: 10.1038/srep33867 (2016).

## References

[b1] O’Shea D., Junge C., Volz J. & Rauschenbeutel A. Fiber-optical switch controlled by a single atom. Phys. Rev. Lett. 111, 193601 (2013). 24266471 10.1103/PhysRevLett.111.193601

[b2] Huang J. F., Shi T., Sun C. P. & Nori F. Controlling single-photon transport in waveguides with finite cross section. Phys. Rev. A 88, 013836 (2013).

[b3] Englund D. . Resonant excitation of a quantum dot strongly coupled to a photonic crystal nanocavity. Phys. Rev. Lett. 104, 073904 (2010). 20366887 10.1103/PhysRevLett.104.073904

[b4] Cheng M. T. & Song Y. Y. Fano resonance analysis in a pair of semiconductor quantum dots coupling to a metal nanowire. Opt. Lett. 37, 978–980 (2012). 22378458 10.1364/OL.37.000978

[b5] Liao J. Q. & Law C. K. Correlated two-photon scattering in cavity optomechanics. Phys. Rev. A 87, 043809 (2013).

[b6] Shen J. T. & Fan S. Strongly correlated two-photon transport in a one-dimensional waveguide coupled to a two-level system. Phys. Rev. Lett. 98, 153003 (2007). 17501344 10.1103/PhysRevLett.98.153003

[b7] Chang D. E., Sørensen A. S., Demler E. A. & Lukin M. D. A single-photon transistor using nanoscale surface plasmons. Nat. Phys. 3, 807–812 (2007).

[b8] Longo P., Schmitteckert P. & Busch K. Few-photon transport in low-dimensional systems. Phys. Rev. A 83, 063828 (2011). 10.1103/PhysRevLett.104.023602 20366595

[b9] Kolchin P., Oulton R. F. & Zhang X. Nonlinear quantum optics in a waveguide: Distinct single photons strongly interacting at the single atom level. Phys. Rev. Lett. 106, 113601 (2011). 21469860 10.1103/PhysRevLett.106.113601

[b10] Zheng H., Gauthier D. J. & Baranger H. U. Strongly correlated photons generated by coupling a three- or four-level system to a waveguide. Phys. Rev. A 85, 043832 (2012).

[b11] Roy D. Two-photon scattering of a tightly focused weak light beam from a small atomic ensemble: An optical probe to detect atomic level structures. Phys. Rev. A 87, 063819 (2013).

[b12] Shi T., Fan S. & Sun C. P. Two-photon transport in a waveguide coupled to a cavity in a two-level system. Phys. Rev. A 84, 063803 (2011).

[b13] Ji Z. & Gao S. Two-photon scattering by a cavity-coupled two-level emitter in a one-dimensional waveguide. Opt. Commun. 285, 1302–1307 (2012).

[b14] Liao J. Q. & Law C. K. Correlated two-photon transport in a one-dimensional waveguide side-coupled to a nonlinear cavity. Phys. Rev. A 82, 053836 (2010).

[b15] Birnbaum K. M. . Photon blockade in an optical cavity with one trapped atom. Nature 436, 87–90 (2005). 16001065 10.1038/nature03804

[b16] Dayan B. . A photon turnstile dynamically regulated by one atom. Science 319, 1062–1065 (2008). 18292335 10.1126/science.1152261

[b17] Lang C. . Observation of resonant photon blockade at microwave frequencies using correlation function measurements. Phys. Rev. Lett. 106, 243601 (2011). 21770569 10.1103/PhysRevLett.106.243601

[b18] Kekatpure R. D., Barnard E. S., Cai W. & Brongersma M. L. Phase-coupled plasmon-induced transparency. Phys. Rev. Lett. 104, 243902 (2010). 20867303 10.1103/PhysRevLett.104.243902

[b19] Huang Y., Min C. & Veronis G. Subwavelength slow-light waveguides based on a plasmonic analogue of electromagnetically induced transparency. Appl. Phys. Lett. 99, 143117 (2011).

[b20] Han Z. & Bozhevolnyi S. I. Plasmon-induced transparency with detuned ultracompact Fabry-Pérot resonators in integrated plasmonic devices. Opt. Express 19, 3251–3257 (2011). 21369147 10.1364/OE.19.003251

[b21] Chen J., Wang C., Zhang R. & Xiao J. Multiple plasmon-induced transparencies in coupled-resonator systems. Opt. Lett. 37, 5133–5135 (2012). 23258029 10.1364/OL.37.005133

[b22] Xiao Y. F. . Asymmetric Fano resonance analysis in indirectly coupled microresonators. Phys. Rev. A 82, 065804 (2010).

[b23] Tu X., Mario L. Y. & Mei T. Coupled Fano resonators. Opt. Express 18, 18820–18831 (2010). 20940775 10.1364/OE.18.018820

[b24] Lu H., Liu X., Mao D. & Wang G. Plasmonic nanosensor based on Fano resonance in waveguide-coupled resonators. Opt. Lett. 37, 3780–3782 (2012). 23041857 10.1364/ol.37.003780

[b25] Dong G., Zhang Y., Kamran M. A. & Zou B. Group delay of single-photon transmission in a waveguide side coupled with a Jaynes-Cummings chain. J. Appl. Phys. 113, 143105 (2013).

[b26] Tan H. T., Zhang W. M. & Li G. X. Entangling two distant nanocavities via a waveguide. Phys. Rev. A 83, 062310 (2011).

[b27] Gullans M., Chang D. E., Koppens F. H. L., de Abajo F. J. G. & Lukin M. D. Single-photon nonlinear optics with graphene plasmons. Phys. Rev. Lett. 111, 247401 (2013). 24483697 10.1103/PhysRevLett.111.247401

[b28] Shi T. & Fan S. Two-photon transport through a waveguide coupling to a whispering-gallery resonator containing an atom and photon-blockade effect. Phys. Rev. A 87, 063818 (2013).

[b29] Xu S., Rephaeli E. & Fan S. Analytic properties of two-photon scattering matrix in integrated quantum systems determined by the cluster decomposition principle. Phys. Rev. Lett. 111, 223602 (2013). 24329447 10.1103/PhysRevLett.111.223602

[b30] Zheng H., Gauthier D. J. & Baranger H. U. Waveguide QED: Many-body bound-state effects in coherent and Fock-state scattering from a two-level system. Phys. Rev. A 82, 063816 (2010).

[b31] Baragiola B. Q., Cook R. L., Brańczyk A. M. & Combes J. *N*-photon wave packets interacting with an arbitrary quantum system. Phys. Rev. A 86, 013811 (2012).

[b32] Chumak O. O. & Stolyarov E. V. Photon distribution function for propagation of two-photon pulses in waveguide-qubit systems. Phys. Rev. A 90, 063832 (2014).

[b33] Nysteen A., Kristensen P. T., McCutcheon D. P. S., Kaer P. & Mørk J. Scattering of two photons on a quantum emitter in a one-dimensional waveguide: Exact dynamics and induced correlations. New J. Phys. 17, 023030 (2015).

[b34] Cheng M. T., Ma X. S., Ding M. T., Luo Y. Q. & Zhao G. X. Single-photon transport in one-dimensional coupled-resonator waveguide with local and nonlocal coupling to a nanocavity containing a two-level system. Phys. Rev. A 85, 053840 (2012).

[b35] Zhang Y. & Zou B. Effects of non-*δ* coupling between one-dimensional waveguides and side optical cavities. Phys. Rev. A 89, 063815 (2014).

[b36] Watts M. R. . Microring-resonator filter with doubled free-spectral-range by two-point coupling. Conference on Lasers and Electro-Optics/Quantum Electronics and Laser Science and Photonic Applications Systems Technologies, Technical Digest (Optical Society of America, 2005).

[b37] Guo X. . Direct coupling of plasmonic and photonic nanowires for hybrid nanophotonic components and circuits. Nano Lett. 9, 4515–4519 (2009). 19995088 10.1021/nl902860d

[b38] Xiao Y. . Single-nanowire single-mode laser. Nano Lett. 11, 1122–1126 (2011). 21322600 10.1021/nl1040308

[b39] Solis J. Jr. . Turning the corner: Efficient energy transfer in bent plasmonic nanoparticle chain waveguides. Nano Lett. 13, 4779–4784 (2013). 24020385 10.1021/nl402358h

[b40] Shen J. T. & Fan S. Theory of single-photon transport in a single-mode waveguide. I. Coupling to a cavity containing a two-level atom. Phys. Rev. A 79, 023837 (2009).

[b41] Brabec T. & Krausz F. Intense few-cycle laser fields: Frontiers of nonlinear optics. Rev. Mod. Phys. 72, 545–591 (2000).

